# Guy’s and St Thomas NHS Foundation active surveillance prostate cancer cohort: a characterisation of a prostate cancer active surveillance database

**DOI:** 10.1186/s12885-021-08255-z

**Published:** 2021-05-19

**Authors:** Salonee Shah, Kerri Beckmann, Mieke Van Hemelrijck, Ben Challacombe, Rick Popert, Prokar Dasgupta, Jonah Rusere, Grace Zisengwe, Oussama Elhage, Aida Santaolalla

**Affiliations:** 1 King’s College London, School of Cancer and Pharmaceutical Sciences, Translational Oncology & Urology Research (TOUR), London, UK; 2 Cancer Research Institute, University of South Australia, Adelaide, Australia; 3 King’s College London, London, UK & Guy’s and St Thomas’ NHS Foundation Trust, London, UK

**Keywords:** Prostate cancer, Active surveillance, Cohort

## Abstract

**Background:**

The routine clinical use of serum prostatic specific antigen (PSA) testing has allowed earlier detection of low-grade prostate cancer (PCa) with more favourable characteristics, leading to increased acceptance of management by active surveillance (AS). AS aims to avoid over treatment in men with low and intermediate-risk PCa and multiple governing bodies have described several AS protocols. This study provides a descriptive profile of the Guy’s and St Thomas NHS Foundation Trust (GSTT) AS cohort as a platform for future research in AS pathways in PCa.

**Methods:**

Demographic and baseline characteristics were retrospectively collected in a database for patients at the GSTT AS clinic with prospective collection of follow-up data from 2012. Seven hundred eighty-eight men being monitored at GSTT with histologically confirmed intermediate-risk PCa, at least 1 follow-up appointment and diagnostic characteristics consistent with AS criteria were included in the profile. Descriptive statistics, Kaplan-Meier survival curves and multivariable Cox proportion hazards regression models were used to characterize the cohort.

**Discussion:**

A relatively large proportion of the cohort includes men of African/Afro-Caribbean descent (22%). More frequent use of magnetic resonance imaging and trans-perineal biopsies at diagnosis was observed among patients diagnosed after 2012. Those who underwent trans-rectal ultrasound diagnostic biopsy received their first surveillance biopsy 20 months earlier than those who underwent trans-perineal diagnostic biopsy. At 3 years, 76.1% men remained treatment free. Predictors of treatment progression included Gleason score 3 + 4 (Hazard ratio (HR): 2.41, 95% Confidence interval (CI): 1.79–3.26) and more than 2 positive cores taken at biopsy (HR: 2.65, CI: 1.94–3.62). A decreased risk of progressing to treatment was seen among men diagnosed after 2012 (HR: 0.72, CI: 0.53–0.98).

**Conclusion:**

An organised biopsy surveillance approach, via two different AS pathways according to the patient’s diagnostic method, can be seen within the GSTT cohort. Risk of patients progressing to treatment has decreased in the period since 2012 compared with the prior period with more than half of the cohort remaining treatment free at 5 years, highlighting that the fundamental aims of AS at GSTT are being met. Thus, this cohort is a good resource to investigate the AS treatment pathway.

**Supplementary Information:**

The online version contains supplementary material available at 10.1186/s12885-021-08255-z.

## Background

Globally, prostate cancer (PCa) is the second most common malignancy in men. With an estimated 1 million new cases in 2018, it has now become the 5th leading cause of cancer death worldwide [1]. Due to the widespread use of serum prostate specific antigen (PSA) testing and extended prostate biopsy techniques, PCa is usually detected at an earlier stage. Hence, tumours tend to have more favourable clinical characteristics and long-term survival outcomes [2]. This has led to the rapid evolution and acceptance of active surveillance (AS) treatment for low and intermediate-risk PCa.

Previously, men with localized PCa would have undergone radical removal of the whole prostate gland leading to a high rate of overtreatment of clinically insignificant PCa (suggested to be as high as 56%) [3]. AS enables close monitoring under an organised regime of PSA testing, biopsies and/or magnetic resonance imaging (MRI) that allows patients to be regularly observed for signs of disease progression, indicating the need for active treatment rather than commencing radical therapy immediately [4]. From a medical perspective, the benefit and effectiveness of AS is well documented [5, 6], and AS has the potential to provide fewer physical symptoms than a radical prostatectomy such as sexual and urinary function symptoms [7]. However, recently more studies have focused on the effects on mental health and well-being throughout the process, especially during biopsies. Uncertainty that arises when living with an untreated cancer has the potential to cause significant emotional burden, increasing a patient’s anxiety [8]. A clear need for a less invasive monitoring approach is highlighting the potential increase in use of MRI instead of biopsies as a PCa diagnostic and monitoring test [9].

While generally accepted as a suitable management option for low risk PCa, AS monitoring regime vary among clinical practices and guidelines, leading to it being questioned by some clinicians and patients [10]. In effort to minimise this, the Movember Foundation launched the Global Action Plan Prostate Cancer Active Surveillance initiative (GAP3) which has combined data from AS cohorts worldwide to create a global consensus for selection, monitoring and treatment intervention thresholds [11]. Most AS protocols at UK centres are based upon NICE and EAU guidelines [12, 13], however current clinical practices at UK centres vary from these guidelines [7]. While Kinsella et al. [5] documents protocols for several published AS cohorts, practices at GSTT are yet to be described in detail. With a relatively long follow up and ethnically diverse patient population, the GSTT AS cohort (GSTT-AS) is now one of the largest participating UK centres within the GAP3 initiative [14]. Monitoring AS cohorts provides an avenue to explore differences between other global surveillance protocols, helping identify standards that define the best surveillance approach for better patient outcomes.

This study profiles the GSTT-AS, part of the wider GAP3 initiative, by characterizing patients undergoing surveillance, follow-up procedures and trends in patient outcomes. Additionally, it aims to identify possible predictive factors for converting to active treatment, and highlights changes in practices over time.

## Construction and content

### Database

GSTT-AS was created in 2012. AS patients who attended the AS clinic at the GSTT Urology centre were identified from clinic lists, and data for baseline characteristics were collected retrospectively, with prospective collection of follow-up data after 2012, until 2020. GSTT-AS is part of Guy’s Cancer Cohort, a research ethics committee approved research database (Reference number: 18/NW/0297) of all routinely collected clinical data of cancer patients at GSTT.

### Data collection

**Fig. 1:**
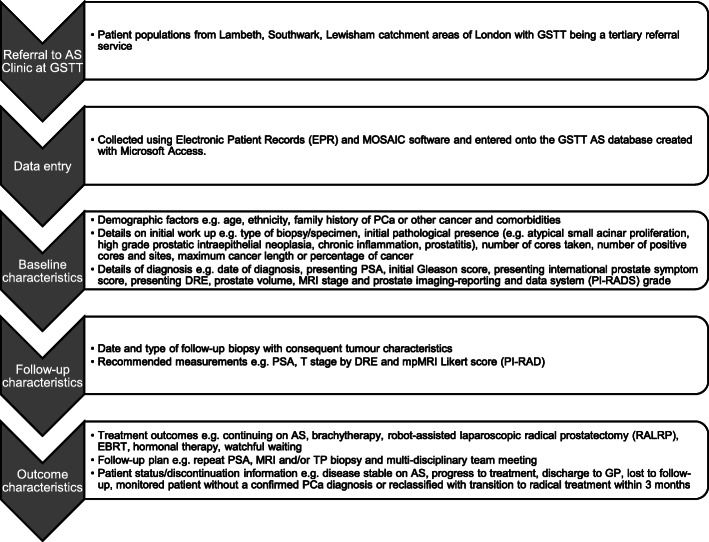
A workflow showing collection of data for GSTT AS database

Data were collected from the Electronic Patient Records (EPR) and the Cancer Information Solution (MOSAIQ) software at GSTT and manually entered into the database. EPR is clinical software, which brings together key clinical and administrative data and allows for letters as well as other important documents to be uploaded, ensuring easy access to a thorough patient history and clinical pathway. MOSAIQ is a care management software for medical oncology allowing aspects of patient cancer data, chemotherapy regimens and pharmacy information to be collected in a common point of access. GSTT-AS database includes three sections: baseline characteristics, follow-up procedures and patient outcomes at last assessment. Baseline data items extracted and recorded in GSTT-AS included patient demographic factors and details on initial work up and diagnosis. AS follow-up data included date of follow-up and type or specimen of biopsy, with the consequent tumour characteristics and recommended measurements recorded. Finally, the treatment outcomes, the follow-up plan and patient status/discontinuation were prospectively updated within GSTT-AS. Figure 1 shows a workflow for the data collection process.

### Study population

GSTT-AS includes patient populations from Lambeth, Southwark and Lewisham catchment areas of London with GSTT being a tertiary referral service. This widespread outreach results in a population with ethnic and socioeconomic diversity. The current criteria for selection for AS at GSTT includes PSA of ≤10 ng/ml (or < 15 ng if age 70 years or more), clinical stage of T1a/T1b if life expectancy > 10 years, T2 well/moderately differentiated disease with life expectancy > 10 years and asymptomatic and Gleason score (GS) ≤ 6 (or ≤ 3 + 4 = 7 if age 70 years or more). Routine multi-parametric MRI and TP biopsies are used for monitoring disease and clinical evidence of disease progression, as well as patient request, warrant a referral for commencing active treatment. GSTT-AS consists of patients diagnosed from 2004 onwards, many of which were included before any formal AS protocols were in place and hence some patients fall outside of the current inclusion criteria. For the purpose of the characterization of the cohort, only patients with a histological diagnosis of PCa, characteristics consistent with being on AS and at least one follow-up appointment were included in the study. Hence the exclusion criteria included age > 80 years, serum PSA > 20 ng/ml, GS > 3 + 4 and T stage >T2 at diagnosis. Patients being who underwent PCa treatment within 3 months of diagnosis or with missing follow-up information were also excluded (Supplementary Figure 1).

### Statistical analysis

Descriptive statistics were used to outline the demographic and clinical characteristics of patients at time of initial PCa diagnosis as well as outcome at last follow-up. The total number of follow-up appointments and mean number of biopsies, MRI and PSA per person were calculated for different demographic subgroups. Subgroup analyses of the cohort before and after 2012 were carried out to identify variations in the cohort and practices according to the diagnostic period, with 2012 corresponding to the timing of GSTT’s more formal adoption of MRI as part of diagnostic and AS pathways. Kaplan-Meier estimation methods were used to determine time to follow-up appointments and re-biopsy, as well as progression to treatment according to clinical characteristics and diagnostic procedures. Multivariable Cox proportional hazards regression was used to identify demographic and clinical characteristics associated with risk of progression to treatment while on AS. All statistical analyses were carried out using Stata/MP 16.1.

### Cohort profile

Demographic and clinical characteristics of the study population are shown in Table 1. The mean age of men at diagnosis was 62 years, with 47.3% being between 60 and 69 years. The majority of the cohort (59.7%) were Caucasian patients, with the next largest group being Black African and Black Caribbean (22.1%). Most patients were diagnosed between 2009 and 2014. Compared with men diagnosed before 2012, there were more non-Caucasian men included in the AS program from 2012 onwards (35.3% vs 25.8%, *p* = 0.04). Also, there was a higher proportion of men with more than three positive cores (22.9 vs 16.5%, *p* = 0.02) and mean presenting PSA levels tended to be slightly higher (*p* = 0.001) among more recently diagnosed men on AS. There were no apparent differences in age, GS or diagnostic method according to diagnostic period, though significantly more men had undergone MRI imaging as part of their diagnostic work up in the latter period.

**Table 1:** Patient characteristics at initial diagnosis for the total population and stratified by year of diagnosis before or after 2012

Characteristics	Frequency			
	***(n = 788)***	Year of diagnosis		***p***-value
		< 2012	> 2012	
**Age at diagnosis (years)**				
Mean (±SD)	62 (±7.6)	61.4 (±7.2)	61.7 (±7.8)	
Median (IQR)	63 (57–67)	63 (57–63)	62 (56–67)	
**Age at diagnosis (years)**				0.86
	**n (%)**			
≤ 65	496 (62.9)	289 (64.1)	207 (64.7)	
> 65	275 (34.9)	162 (35.9)	113 (35.3)	
< 50	59 (7.5)	–	–	
50–59	219 (27.7)	–	–	
60–69	374 (47.3)	–	–	
70–80	119 (15.0)	–	–	
Unrecorded	17 (2.2)	–	–	
**Year of Diagnosis**	**n (%)**	457 (58.0)	331 (42.0)	**–**
< 2009	107 (13.5)	–	–	
2009–2011	261 (33.0)	–	–	
2012–2014	296 (37.4)	–	–	
2015–2018	124 (15.7)	–	–	
**Ethnicity**	**n (%)**	*n = 669*		0.04
Caucasian	472 (59.7)	319 (74.2)	152 (63.6)	
Black African	107 (13.5)	59 (13.7)	48 (20.1)	
Black Caribbean	69 (8.6)	40 (9.3)	29 (12.1)	
Asian	22 (2.8)	12 (2.8)	10 (4.2)	
Unrecorded	119 (15.1)	–	–	
**PSA level**	**(ng/mL)**			0.001
Mean (±SD)	6.6 (± 3.4)	6.4 (±3.3)	6.9 (±3.5)	
Median (IQR)	6 (4.4–8.0)	6.0 (4.0–8.7)	6.29 (4.7–8.2)	
**PSA level (ng/mL)**	**n (%)**	*n = 718*		0.09
0–5	273 (34.6)	173 (40.8)	100 (34.0)	
5 .1–10	350 (44.4)	193 (45.5)	157 (53.4)	
10.1–15	74 (9.4)	48 (11.3)	26 (8.8)	
15.1–20	21 (2.7)	10 (2.4)	11 (3.7)	
Unrecorded	70 (8.9)	–	–	
**T Stage (at DRE)**	**n (%)**			
Benign	331 (42.0)	–	–	
T2	176 (22.3)	–	–	
**Number having a diagnostic MRI**	**n (%)**			0.001
MRI at diagnosis	235 (29.8)	64 (14.0)	171 (51.7)	
Unrecorded	553 (70.2)	393 (86.0)	160 (48.3)	
**Diagnostic MRI T Stage**	*n = 235*			–
	**n (%)**			
T0	24 (10.2)	–	–	
T2	135 (57.4)	–	–	
Unrecorded	76 (32.3)	–	–	
**Method of diagnostic biopsy**	**n (%)**	*n = 708*		0.12
TP	254 (32.2)	141 (33.6)	113 (39.2)	
TRUS	422 (53.6)	263 (62.6)	159 (55.2)	
Other^a^	32 (4.1)	16 (3.8)	16 (5.6)	
Unrecorded	80 (10.2)	–	–	
**Gleason Score**	**n (%)**	*n = 730*		0.75
≤ 3 + 3	595 (75.5)	357 (81.9)	238 (81.0)	
3 + 4	135 (17.1)	79 (18.1)	56 (19.1)	
Unrecorded	58 (7.4)	–	–	
**Number of cores taken**				
Mean (±SD)	21.0 (±10.7)	–	–	
Median (IQR)	15 (12–32)	–	–	
**Number of cores taken**				
	**n (%)**	*n = 564*		*0.08*
< 12	37 (4.7)	19 (5.6)	18 (6.8)	
12–14	266 (33.8)	141 (41.3)	125 (47.0)	
15–24	80 (10.2)	49 (14.4))	31 (11.7)	
> 25	224 (28.4)	132 (38.4)	92 (34.6	
Unrecorded	181 (23.0)	–	–	
**Number of positive cores**				
Mean (±SD)	2.3 (±2.1)	–	–	
Median (IQR)	2 (1–3)	–	–	
**Number of positive cores**	***n (%)***	*n = 611*		*0.02*
1	288 (36.6)	178 (52.4)	110 (40.6)	
2	129 (16.4)	64 (18.8)	65 (22.9)	
3	76 (9.6)	64 (12.4)	34 (12.6)	
> 3	118 (15.0)	56 (16.5)	62 (22.9)	
Unrecorded	177 (22.5)	–	–	

Statistical test: Pearson’s Chi squared

*PSA* prostate specific antigen, *MRI* magnetic resonance imaging, *DRE* digital rectal exam, *TP* trans-perineal, *TRUS* trans-rectal ultrasound, *SD* standard deviation, *IQR* interquartile range

^a^Other = HOLEP, TURP

**Fig. 2:**
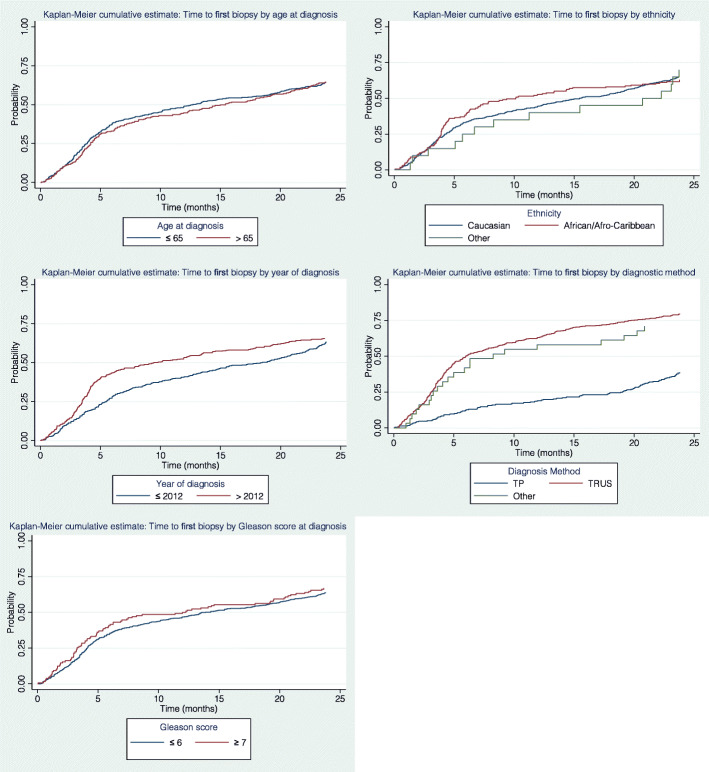
Kaplan-Meier cumulative estimates for time taken to first biopsy on AS by different characteristics at diagnosis. TP – trans-perineal, TRUS = trans-rectal ultrasound, Other – HoLEP/Transurethral resection of the prostate

Table 2 and Fig. 2 summarize the follow-up time and characteristics of biopsy and MRI procedures undertaken during follow-up appointments among men on AS. Twenty percent of the cohort had not undergone any follow-up biopsy procedures while almost half (45.8%) had undergone one biopsy and 35% had undergone two or more biopsies whilst on AS and the median time from diagnosis to the first follow-up biopsy was 13 months. Patients who had a TRUS biopsy at diagnosis tended to receive their first follow-up biopsy around 6 months post diagnosis, which was on average 20 months before those who has a TP biopsy at diagnosis. 66.5% of patients had between one and three MRIs and the median time to the first MRI was 3.8 years from diagnosis. The interval to first MRI differed according to the patient’s original diagnostic method, with those who had undergone a TRUS diagnostic biopsy having had their first MRI an average of 9 months earlier than those who had undergone a TP diagnostic biopsy.

**Table 2:** A summary of the follow-up characteristics of the GSTT-AS cohort

Characteristics	Frequency *(n = 788)*
**No. of years on AS**	
Mean (±SD)	4.7 (±3.06)
Median (IQR)	4.3 (2.5–6.5)
**Number of follow-up biopsies per person**	***n (%)***
0	160 (20.3)
1	361 (45.8)
2	210 (26.7)
> 3	57 (7.2)
**Median time to first follow-up biopsy**	***Months (95% CI)***
Biopsy follow up time	13 (11.6–16.7)
**Median time to first follow-up biopsy by diagnostic method**	***Months (95% CI)***
TP	26 (25–27)
TRUS	6 (5–8)
Other	8 (4–20)
**Number of follow-up MRIs per person**	*n (%)*
0	237 (30.1)
1	297 (37.7)
2	153 (19.4)
> 3	101 (12.8)
**Median time to first follow-up MRI**	***Months (95% CI)***
MRI follow up time	45 (37–53)
**Median time to first follow-up MRI by diagnostic method**	***Months (95% CI)***
TP	52 (33–60)
TRUS	43 (36–53)
Other	28 (6–63)

*AS* active surveillance, *TP* trans-perineal, *TRUS* trans-rectal ultrasound, *MRI* magnetic resonance imaging, *SD* standard deviation, *IQR* interquartile range

Table 3  presents  outcomes for patients at 3 and 5 years on AS. The majority of the cohort (67.4%) remained on AS, at 3 years while 40.6% remained at 5 years. The proportions of men who had transitioned to treatment by 3 and 5 years were 21.7 and 30.1% respectively.

**Table 3:** Patient outcomes at 3 and 5 years

Characteristics	Frequency
**Patient outcome at 3 years**	***n (%), n = 722***
Still on AS	531 (67.4)
Progress to treatment	171 (21.7)
Discharged to GP/WW	19 (2.4)
Lost to follow-up	1 (0.1)
*Censored (n = 788)*	66 (8.4)
**Patient outcome at 5 years**	***n (%), n = 610***
Still on AS	320 (40.6)
Progress to treatment	237 (30.1)
Discharged to GP/WW	49 (6.2)
Lost to follow-up	4 (0.5)
*Censored (n = 788)*	*178 (22.6)*

*AS* active surveillance, *WW* watchful waiting

**Fig. 3:**
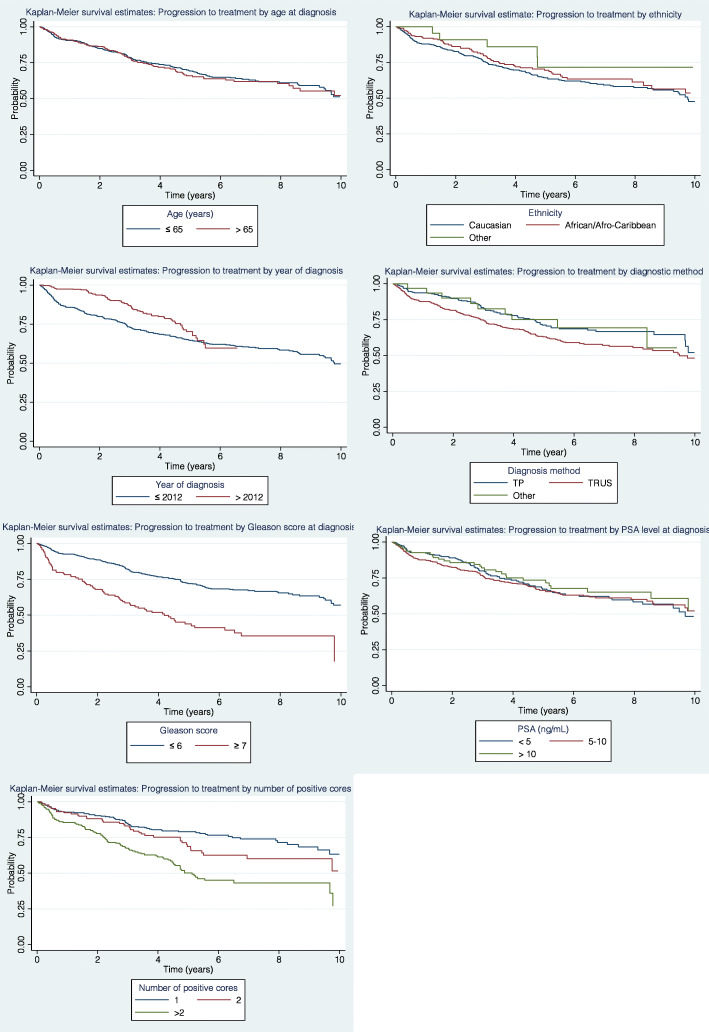
Kaplan-Meier curves for progression to treatment by different characteristics at diagnosis. TP – trans-perineal, TRUS = trans-rectal ultrasound, Other – HoLEP/Transurethral resection of the prostate

The Kaplan-Meier survival curves present treatment-free proportions over time since initial diagnosis, according to different patient characteristics (Fig. 3). Within the first 5 years of follow-up, patients diagnosed after 2012 were more likely to be treatment-free compared to those diagnosed after 2012. Treatment-free progression was also more likely among those who underwent TP diagnostic biopsy. Men with GS 3 + 4 had a higher risk of progressing to treatment compared with patients with GS ≤3 + 3, as do those with an increasing number of positive cores.

Table 4 shows hazard ratios for risk of conversion to treatment, derived from multivariable Cox proportion Hazards regression. Lower risk of conversion was evident for patients diagnosed after 2012 compared with those diagnosed earlier (HR: 0.72, 95% CI: 0.53–0.98). Having GS 3 + 4 compared with 3 + 3 at diagnosis (HR: 2.41, CI: 1.79–3.26) and two or more positives cores compared with one positive core at diagnosis (HR: 2.65, CI 1.94–3.62) independently predicted risk of progression to treatment.

**Table 4:** Showing hazard ratios for risk of progression to treatment, by different patient characteristics and clinical information at diagnosis (from multivariable cox regression)

Characteristics	Hazard Ratio (95% CI)
**Age at diagnosis (years)**	
≤ 65	Reference
> 65	0.89 (0.68–1.17)
**Year of diagnosis**	
≤ 2012	Reference
> 2012	0.72 (0.53–0.98)
**Ethnicity**	
Caucasian	Reference
African/Afro-Caribbean	0.86 (0.64–1.15)
Other	0.54 (0.22–1.31)
**PSA level (ng/mL)**	
< 5	Reference
5–10	1.04 (0.78–1.38)
> 10	0.82 (0.53–1.29)
**Diagnostic Method**	
TP	Reference
TRUS	1.25 (0.85–1.83)
Other	0.91 (0.41–2.00)
**Diagnostic MRI**	
No MRI at diagnosis	Reference
MRI at diagnosis	0.75 (0.54–1.03)
**Gleason Score**	
≤ 3 + 3	Reference
3 + 4	2.41 (1.79–3.26)
**Number of cores taken**	
< 12	Reference
12–14	0.86 (0.49–1.49)
15–24	0.96 (0.49–1.89)
> 25	1.16 (0.58–2.34)
**Number of positive cores**	
1	Reference
2	1.44 (0.98–2.13)
> 2	2.65 (1.94–3.62)

*CI* confidence interval, *PSA* prostate specific antigen, *TP* trans-perineal, *TRUS* trans-rectal ultrasound, *MRI* magnetic resonance imaging

## Utility and discussion

With focus on baseline characteristics, details of follow-up, clinical outcomes and changes in practice over time, our cohort description has identified some specific characteristics that make the GSTT-AS a useful resource for clinical research focused on AS for PCa.

It is interesting to note that there was a relatively large proportion of men of African/Afro-Caribbean descent. This may in part explain the inclusion of some men with higher PSA levels in the GSTT AS cohort, given that high PSA levels at diagnosis have been observed among Black African/Caribbean men previously [15, 16]. The diversity of the GSTT cohort provides opportunities to explore variations in AS patient pathway and their outcomes according to ethnicity. However, our results showed no significant difference in the risk of conversion to treatment in different ethnic groups. Beckmann et al. explored this further and investigated protocol adherence and outcomes among African/Afro-Caribbean males at GSTT [17]. Similar findings were observed and the need for a more tailored program with careful monitoring for this cohort of men was indicated. Our cohort also includes men who fall outside NICE and EAU guidelines for AS eligibility, including 12% with PSA > 10 ng/ml and 15% with more than three positive cores at diagnosis [12, 18]. Whilst men with PSA > 10 ng/ml do not appear to have increased risk of converting to treatment, the latter may be at greater risk of progressing to treatment and therefore reflecting the greater use of TP biopsy and MRI for men enrolled in AS in more recent years.

At GSTT, men diagnosed after 2012 were more likely to receive MRI scans at diagnosis (51.7% of men > 2012 in vs. 14.0% < 2012). In 2015, NICE released a guideline encouraging the use of an early mpMRI for the initial assessment of candidates for AS, however GSTT protocols adopted the use of MRI in diagnosis and follow-up earlier, through the evidence and guidelines available circa 2012. The European Society of Urogenital Radiology (ESUR) published guidelines for mpMRI in 2012 and stated that a TRUS biopsy underestimates the extent and grade of PCa [19]. PROMIS (PROstate MRI Imaging Study) also found that mpMRI has the potential to outperform the accuracy of a TRUS biopsy in PCa diagnosis [9]. When TRUS biopsies were then guided by mpMRI findings, an increase of 18% in the detection of clinically significant cancers was observed [9]. The release of the ESUR guidelines, plus the trial evidence indicating the benefits of MRI may be the reason for more patients at GSTT having MRI at diagnosis after 2012. Further research is required to assess whether early adoption of MRI within the GSTT AS protocol has led to more accurate diagnoses of clinically significant and insignificant PCa resulting in better selection of men for AS.

Increased use of a TP diagnostic biopsy since 2012 at GSTT was observed (39.2% > 2012 vs. 33.6% < 2012, *p* = 0.12). Whilst NICE guidance acknowledges the different biopsy techniques available, data on the outcomes of TRUS diagnostic biopsy are more widely available and so remain the recommended diagnostic biopsy method [12]. However, determining the most beneficial biopsy method is a controversial topic in current literature. Studies comparing TRUS and TP detection and complication rates remain inconclusive [20, 21], though the recent meta-analysis by Xiang et al. found that both methods had similar diagnostic efficacy but that TP biopsy was associated with a significant decrease in infection and rectal bleeding [22]. Historically, TP biopsy required the use of general anaesthetic and extensive histological analysis, however researchers at GSTT have recently investigated the outcomes of the PrecisionPoint™ access system (Perineologic, Cumberland, MD, USA) for freehand TP biopsies which are less resource intensive than conventional TP biopsies as they can be carried out under local anaesthetic [22]. Two distinct pathways of AS follow-up can also be seen within the GSTT-AS. When diagnosed using a TRUS biopsy, median time to the first biopsy was 6 months with 63% of men having the first biopsy within the first year. Men who underwent TP diagnostic biopsy received their first follow-up biopsy much later (median = 26 months) with only 19% having been biopsied within the first year of AS. These findings are in line with current GSTT AS protocols which recommend a confirmatory TP biopsy at 6 months for men initially diagnosed via TRUS biopsy, many of which have been referred to GSTT from other centres. Future directions of this research involve a longer patient follow-up and an assessment of the cost effectiveness of TP biopsy under local anaesthetic vs the gold standard TRUS biopsy.

The differences in the median time to the first MRI (45 months) and first biopsy (13 months) during follow-up at GSTT show that some patients underwent biopsies without a previous MRI. Due to the non-invasive nature, MRI has sparked interest in its serial use during follow-up of men on AS in many healthcare systems and from 2014, NICE guidelines encouraged the use of mpMRI as part of AS follow-up to detect disease progression and drive repeat biopsies [12]. However findings in studies investigating the role of serial MRI for AS monitoring are contradictory [23–25]. The Prostate Cancer Radiological Estimation of Change in Sequential Evaluation (PRECISE) panel have developed recommendations in effort to highlight clearer thresholds identifying radiologically significant disease progression [26]. Since GSTT-AS spans diagnoses from 2002 onwards, many of the cohort would have been monitored before MRI-guided biopsy became a recommended part of follow-up, with our results reflecting changes to the GSTT protocol over time. Further investigations with longer follow-up of patients enrolled after MRI was incorporated into the GSTT AS protocol are required to assess its benefits.

In line with the findings from Royal Marsden and Toronto cohort, our cohort shows GS of 3 + 4 to be highly predictive for progressing to treatment (HR: 2.41 CI: 1.79–3.26) when compared with GS 3 + 3 [27, 28]. However, many of the patients in older studies, such as the Toronto cohort, were graded using an older version of the Gleason grading system (1996–2005). Re-grading was not performed resulting in different stratification of low and intermediate risk groups with many of the patients graded GS 6 possibly being re-graded to GS 3 + 4 using the modified Gleason system [29]. Currently, the suitability of men with GS 3 + 4 for AS is still debated [30, 31]. GSTT-AS only contains a small number of patients diagnosed before 2006 and hence the majority are classified according to more updated versions of Gleason grading criteria [29] making it a useful resource for the scientific community for further research to refine GS threshold for entry onto active surveillance.

A significant increased risk of progression to treatment in GSTT-AS was observed for patients with more than two positive cores compared with one positive core as has been observed in other cohorts [27, 31]. Whilst the number of positive cores has been described to be a positive predictor for progression to treatment, the pathway for patients with a negative biopsy on follow-up is unclear. Using data from GSTT-AS our team has recently investigated the possibility of modifying AS protocols for men with negative findings on prostate re-biopsy [32].

Our finding of lower risk of conversion to active treatment among patients diagnosed after 2012 translates into fewer men converting to radical treatment, hence less potential for side effects such as urinary incontinence and erectile dysfunction [13]. This finding could indicate better selection of candidates for AS at entry can lead to better outcomes and therefore supports the rationale behind the GSTT protocol since 2012, with its increased use of MRI scans and TP biopsy at diagnosis, and the increased number of cores taken. Verification that these strategies decrease the risk of conversion to treatment in the longer term requires further investigation, ideally in a trial setting [33].

### Limitations

The data collection was mainly retrospective which allows for the introduction of selection and information bias. During the initial start-up of GSTT-AS not all AS patients were enrolled, resulting in interrupted patient selection. Given the nature of the cohort, only the information available in the patient record can be retrieved limiting the collection of information on confounders such as BMI and comorbidities. Data collected on imaging methods during follow-up should be a focus of ongoing and future studies. Efforts should be made to establish prospective data collection so that up-to-date information can be shared with researchers and clinicians to help guide the AS management strategy.

## Conclusion

In an effort to reduce overtreatment in patients with clinically insignificant PCa, different AS protocols have been applied at different centres over the past two decades. The protocol at GSTT, which has evolved over time, differs from some others with its increased use of MRI and TP biopsy with a higher number of cores taken at diagnosis. Two distinct AS follow-up pathways which differed according to the patient’s diagnostic method were evident, with those who underwent a TRUS diagnostic biopsy having their first surveillance biopsy at 6 months and those who underwent a TP diagnostic biopsy receiving their first surveillance biopsy on average 2 years after diagnosis. GSTT-AS contains a unique ethnically diverse population compared with other UK and European cohorts and thus offers future research opportunities exploring aspects of surveillance protocols that could result in better outcomes for men currently on, or considering, AS for PCa.

## Supplementary Information

**Additional file 1: Supplementary figure 1**. A diagrammatic representation of the inclusion criteria for the final analytic cohort

## Data Availability

The data that support the findings of this study are under the cancer cohort approval and data access is granted via request to Guy’s cancer cohort study access committee.

## Abbreviations

AS: Active surveillance
CI: Confidence interval
DRE: Digital rectal exam
EAU: European Association of Urology
EBRT: External beam radiotherapy
GS: Gleason score
GSTT: Guy’s and St Thomas NHS Foundation Trust
GSTT-AS: Guy’s and St Thomas NHS Foundation Trust active surveillance cohort
HR: Hazard ratio
IQR: Interquartile range
KCH: King’s College Hospital
mpMRI: Multi-parametric magnetic resonance imaging
MRI: Magnetic resonance imaging
NICE: National Institute for Health and Care Excellence
PCa: Prostate cancer
PIN: Prostatic intraepithelial neoplasia
PSA: Prostate specific antigen
RALRP: Robot-assisted laparoscopic radical prostatectomy
RP: Radical prostatectomy
SD: Standard deviation
TP: Trans-perineal
TRUS: Trans-rectal ultrasound
WW: Watchful waiting
