# Correction to: Guy’s and St Thomas NHS Foundation active surveillance prostate cancer cohort: a characterisation of a prostate cancer active surveillance database

**DOI:** 10.1186/s12885-022-09188-x

**Published:** 2022-01-24

**Authors:** Salonee Shah, Kerri Beckmann, Mieke Van Hemelrijck, Ben Challacombe, Rick Popert, Prokar Dasgupta, Jonah Rusere, Grace Zisengwe, Oussama Elhage, Aida Santaolalla

**Affiliations:** 1 King’s College London, School of Cancer and Pharmaceutical Sciences, Translational Oncology & Urology Research (TOUR), London, UK; 2 Cancer Research Institute, University of South Australia, Adelaide, Australia; 3 King’s College London & Guy’s and St Thomas’ NHS Foundation Trust, London, UK

**Correction to: BMC Cancer 21, 573 (2021)**

**https://doi.org/10.1186/s12885-021-08255-z**

Following publication of the original article [1], the authors reported an omission from the funding statement. The correct funding statement is supplied below:

This work was supported by the department of Translational Oncology and Urology Research (TOUR) at King’s College London, Medical Research Council Grant no: MR/R014043/1. The views expressed are those of the author(s) and the funding body had no role in the design of the study and collection, analysis, and interpretation of data in writing the manuscript. Kerri Beckmann is supported by national health and medical research council (NHMRC) ECR fellowship GNT 1124210.

## References

[1] Shah S, Beckmann K, Van Hemelrijck M (2021). Guy’s and St Thomas NHS Foundation active surveillance prostate cancer cohort: a characterisation of a prostate cancer active surveillance database. BMC Cancer.

