# Supplementary material for: Guy’s and St Thomas NHS Foundation active surveillance prostate cancer cohort: a characterisation of a prostate cancer active surveillance database
Source: BMC Cancer. 2021 May 19;21:573. doi: 10.1186/s12885-021-08255-z (PMC8135162; doi:10.1186/s12885-021-08255-z)
Supplement: Supplementary file 1 — Additional file 1: Supplementary figure 1. A diagrammatic representation of the inclusion criteria for the final analytic cohort [file 12885_2021_8255_MOESM1_ESM.docx]

*Supplementary figure 1: A diagrammatic representation of the inclusion criteria for the final analytic cohort*

**Number of AS clinic attendees in AS database
(n = 1072)**

**Number of actual PCa cases in AS database**

**(n = 926)**

**Number attending AS clinic at GSTT with PCa**

**(n = 867)**

Records excluded – treated at KCH, no follow-up appointment or had other PCa treatment
(n = 59)

Records excluded – characteristics not in line with AS criteria
(n = 79)

Age>80 (n=9)
PSA>20 (n=38)
GS>3+4 (n=19)
T3 on MRI (n=13)

**Number in final analytic cohort**

**(n = 788)**

Records excluded - no prostate cancer
(n = 146)
